# Prognostic features for quality of life after radical cystectomy and orthotopic neobladder

**DOI:** 10.1590/S1677-5538.IBJU.2015.0491

**Published:** 2016

**Authors:** Alexander Kretschmer, Tobias Grimm, Alexander Buchner, Christian G. Stief, Alexander Karl

**Affiliations:** 1 Department of Urology, Ludwig - Maximilians - University, Munich, Germany

**Keywords:** Quality of Life, Cystectomy, Urinary Bladder Neoplasms, Urinary Incontinence

## Abstract

**Purpose:**

To analyse prognostic features on quality of life (QoL) following radical cystectomy and urinary diversion via orthotopic neobladder in a single-centre patient cohort.

**Materials and Methods:**

Postoperative QoL of 152 patients was assessed retrospectively using the validated QLQ-C30 questionnaire. Potential associations of patient's quality of life including pre-and intraoperative characteristics, surgeon experience, postoperative time course, adjuvant therapies, and functional outcome were defined a priori and evaluated. Mann-Whitney-U-, Kruskal-Wallis-, Spearman correlation and post hoc-testing were used. A multivariate analysis using a multiple logistic regression model was performed. A p value <0.05 was considered to be statistically significant.

**Results:**

Median follow-up was 48 months. Univariate analysis of prognostic features for health-related QoL revealed a significant impact of gender (p=0.019), performance status (p<0.001), experience of surgeon (>100 previous cystectomies, p=0.007), and nerve-sparing surgery (p=0.001). Patients who underwent secondary chemotherapy or radiotherapy had significant lower QLQ-C30 scores (p=0.04, p=0.02 respectively). Patients who were asymptomatic had a significantly higher quality of life (p<0.001). A significant impact of severity of incontinence based on ICIQ-SF score (p<0.001) and daily pad usage (p<0.001), existence of daytime incontinence (p<0.001), existence of urgency symptoms (p=0.007), and IIEF-5 score (p<0.001) could be observed. In multivariate analysis, independent prognostic relevance could be confirmed for preoperative ECOG performance status of 0 (p=0.020 vs. ECOG 1, p=0.047 vs. ECOG 2), experience of the respective surgeon (≥100 vs. <100 previous cystectomies, p=0.021), and daytime continence (p=0.032).

**Conclusion:**

In the present study, we report health-related QoL outcomes in a contemporary patient cohort and confirm preoperative ECOG status, surgeon experience and daytime incontinence as independent prognostic features for a good postoperative QoL.

## INTRODUCTION

Bladder cancer is the second most common malignancy in the urinary tract and transitional cell carcinoma of the urinary bladder is currently the fourth most common malignancy in men in the Western world (1). Neoadjuvant chemotherapy, followed by radical cystectomy, extended pelvic lymphadenectomy, and urinary diversion (using an ileal conduit or ileal orthotopic neobladder) is the gold standard therapeutic approach for muscle invasive bladder cancer (2). However, radical cystectomy comes along with quite a burden for the patient considering a long-term morbidity-rate of up to 90% leading to a considerable mortality rate (3–5). Recently, there was an increasing interest in QoL aspects for patients undergoing radical cystectomy, mostly regarding different types of urinary diversion (6). While short-term and long-term complications and long-term functional outcomes after urinary diversion via orthotopic neobladder have been extensively analysed there is still a lack of data concerning QoL aspects. Functional outcomes have been described to significantly influence the patient's QoL after orthotopic urinary diversion, but little is known about further possible influencing factors (7). Thus, the aim of this study is to investigate various potential contributing factors on patient's QoL after radical cystectomy and urinary diversion via orthotopic neobladder.

## MATERIALS AND METHODS

### 

#### Patient population, study design and data assessment

In one tertiary care centre, 301 cystectomies with urinary diversion via orthotopic neo-bladder were performed between May 2004 and September 2014 by a total number of 14 surgeons. During follow-up period, 92 patients had died. After approval by an institutional review board, data assessment was performed retrospectively via standardized questionnaires that were sent to 209 patients who underwent radical cystectomy and urinary diversion via orthotopic neobladder due to malignancy. Questionnaires were returned by 152 patients and underwent further analysis. This leads to a global response rate of 50.5%.

Health-related QoL was measured using the standardized EORTC QLQ-C30 (global health status; validated German version) (8). The global health status was calculated following current EORTC instructions: score=100* [(question29+question30)/2–1]/6 (9). This indicates that higher scores are associated with an increased QoL.

Potential predefined prognostic features of patient's QoL were evaluated. Hereby we focused on various categories including preoperative characteristics (gender, preoperative age, performance status), intraoperative features (surgeon's experience, nerve sparing operation, operation duration, blood loss, blood transfusion, postoperative revision, postoperative complications), TNM classification, functional outcome (continence status, urgency symptoms, sexual function), adjuvant therapies (chemotherapy, radiotherapy), and symptoms (any symptoms, night sweats, weight loss, flank pain).

The preoperative performance status was determined based on the respective Eastern Cooperative Oncology Group performance (ECOG) status score (10).

Daytime continence was determined using the validated International Consultation on Incontinence Questionnaire (ICIQ) short-form scoring system in a validated German translation, and pad usage (11). The existence of urgency symptoms was assessed. Depending on the respective ICIQ-SF, patient's stress urinary incontinence was classified as mild (1–5), moderate (6–10), and severe (>10) as previously described (11).

Sexual function was assessed using the abridged International Index of Erectile Function (IIEF-5) (12) and abridged Female Sexual Function Index (FSFI-6) (13) respectively. A cut-off score of >20 (out of 25) points was used as the definition of potency as previously described (12). Female sexual dysfunction was defined by a FSFI-6 of less than 18 as previously described by Isidori et al. (13).

### Statistical analysis

Primary endpoint was the QoL (global health status based on EORTC QLQ-C30 questions 29 and 30) of the respective patient. Secondary endpoints were above-mentioned prognostic features. Analysis was performed using the global health status as a continuous variable as well as using a cut-off score of 70 as recently described by Snyder et al. (14). Hereby, a QLQ-C30 score of less than 70 is associated with a poor quality of life, a score of at least 70 is considered to be associated with a good quality of life.

To analyse and compare QoL in the respective subgroups, Mann-Whitney-U-, Kruskal-Wallis-ANOVA, and chi-squared-Test, Spearman's rank correlation, and post-hoc-testing were used whenever indicated. Additionally, a multiple logistic regression model that included all potential prognostic features that showed significant results in the univariate analysis was performed. All statistical analyses were performed using STATISTICA 10 software (StatSoft, Tulsa, OK, USA). A p value <0.05 was considered to be statistically significant.

## RESULTS

### 

#### Pre-and perioperative patient characteristics

Median follow-up, defined as time between radical cystectomy and answering of the questionnaire, was 48 months [3-108]. Follow-up was within the first 12 months in 22% (n=33) of the patient collective. Median patient age was 71 years [44-88 years]. Patient characteristics of the patients that underwent further analysis are summarized in Table-1.

**Table 1:** Baseline patients characteristics, histopathologic results, and stated symptoms following radical cystectomy and orthotopic urinary diversion after a median follow-up of 48 months.

**Gender**		
	Male	87.5% (133/152)
	Female	12.5% (19/152)
**Age at follow-up (years)**		
	Median	71 (range 44 to 88)
**pT-stage**		
	pTis	22.4% (34/152)
	pT1	23.0% (35/152)
	pT2	27.6% (42/152)
	pT3	18.4% (28/152)
	pT4	4.6% (7/152)
**Lymphadenectomy performed**		
	Yes	91.4% (139/152)
	No	8.6% (13/152)
**pN-stage**		
	pN0	89.2% (15/139)
	pN+	10.8% (15/139)
**Symptoms at follow-up**		
	Yes	34.2% (50/146)
	No	65.8% (96/146)
	Unknown	3.9% (6/152)
	Flank pain	15.1% (22/146)
	Night sweats	6.2% (9/146)
	Weight loss	4.1% (6/146)
	Other	8.9% (13/146)

#### Analysis responder vs. non-responder

To analyse for a potential selection bias, we compared preoperative characteristics and pathological tumour stage for those patients who responded to the questionnaire and those who did not respond. Among the patients who did not respond to the questionnaire, 56% were deceased. Median age was 65 years for both groups (p=0.400). Regarding the non-responder group, 48% had a locally advanced disease (pT3-4) (responder: 25%, p<0.001), and 30% had lymph node infiltration during cystectomy (responder: 12%, p<0.001). There were no significant differences regarding the ratio of high-grade tumours (non-responder: 90% vs. responder: 86%, p=0.359), metastatic disease (9% vs. 9%, p=0.971), and (concomitant) carcinoma in situ (39% vs. 48%, p=0.108).

#### Functional outcome

Median ICIQ-SF within our patient collective was 10 (1–21). Depending on the respective ICIQ-SF scores, incontinence was classified as severe in 44% (n=67) of our study group. Regarding pad usage, 40% (n=61) were in need of more than 2 pads daily. Incontinence results are summarized in Table-2.

**Table 2:** Functional outcome following radical cystectomy and orthotopic urinary diversion after a median follow-up of 48 months.

**ICIQ-SF at follow-up**		
	Median	10 (range 4 to 21)
**Severity of incontinence based on IcIQ-sf**		
	Mild (1 - 5)	16.4% (25/152)
	Moderate (6 - 10)	37.5% (57/152)
	Severe (>10)	44.1% (67/152)
	Dry	2.0% (3/152)
**Pad usage at follow-up**		
	0-1	37.5% (57/152)
	2	22.3% (34/152)
	>2	40.1% (61/152)
**IIEF-5 at follow-up**		
	Median	3 (range 1 to 24)
	Unknown	5.2% (7/133)
	>20	5.6% (7/126)
	≤20	94.4% (119/126)
**FSFI-6 at follow-up**		
	Median	4 (range 1 to 20)
	Unknown	21,1% (4/19)
	>18	13,3% (2/15)
	<18	86.7% (13/15)

**ICIQ-SF** = International Consultation on Incontinence Questionnaire short-form; **IIEF-5** = International Index of Erectile Function; **FSFI-6** = abridged Female Sexual Function Index

Data concerning sexual function was available for 126 out of 133 male patients and for 15 out of 19 female patients. Analysis of the respective IIEF-5 scores showed a median IIEF-5 of 3 (1–24) within our study collective. Regarding the female patients, median FSFI-6 was 4 (1–20). Sexual function results are summarized in Table-2.

#### Univariate analysis of prognostic features for health-related QoL

#### Preoperative characteristics

**Figures 1:**
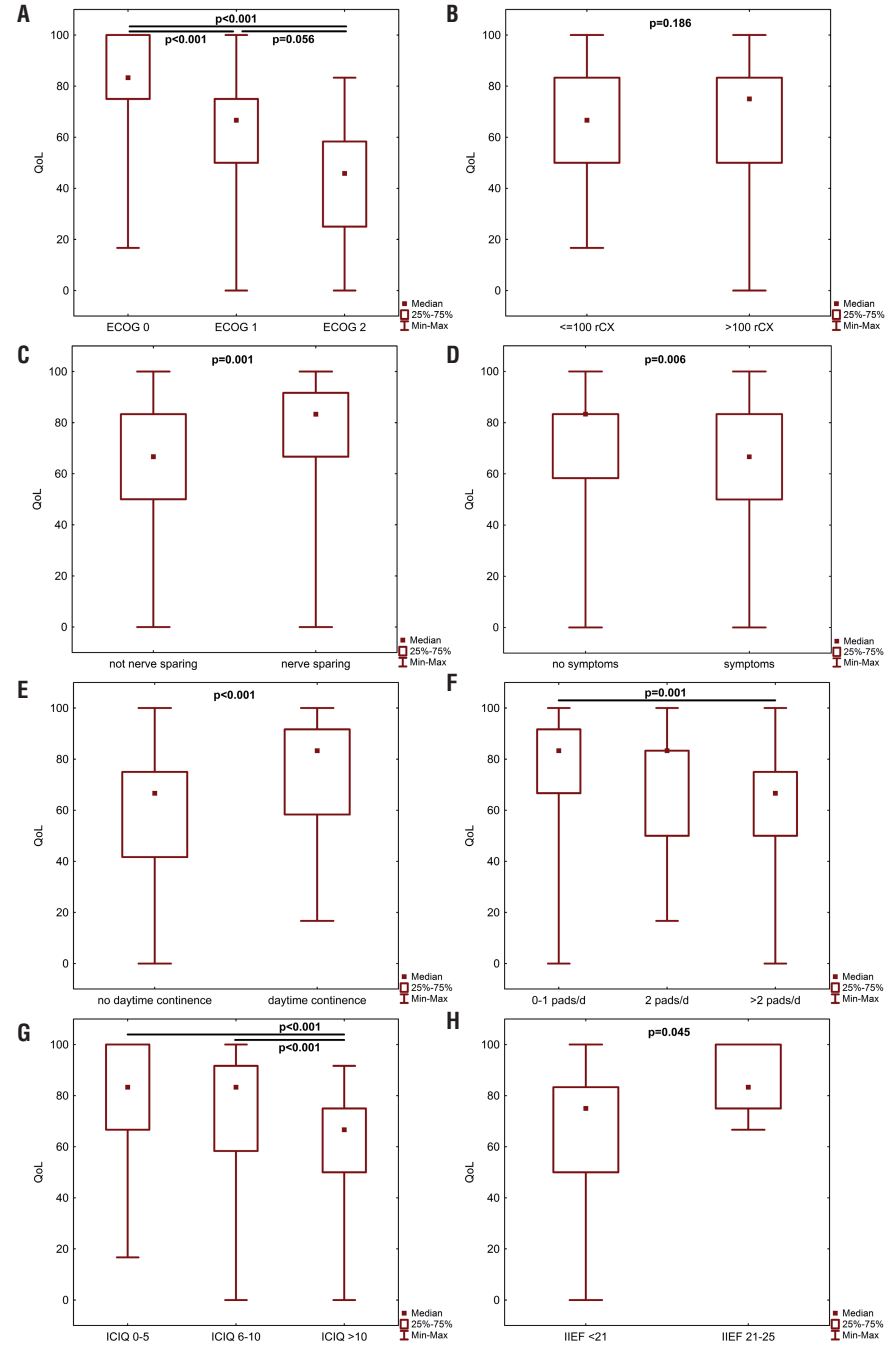
A-H – univariate analysis of multiple predefined prognostic features on postoperative QoL after radical cystectomy and orthotopic urinary diversion. A p value <0.05 was considered statistically significant (**EcOg**=Eastern Cooperative Oncology Group performance status score; **IcIQ-sf**=International Consultation on Incontinence Questionnaire short-form; **IIEf-5**=International Index of Erectile Function; **QoL**=quality of life; **rcx**=radical cystectomy).

At median follow-up, overall health-related QLQ-C30 was 75 for male patients and 67 for female patients (p=0.019). Patients with less or equal 71 years (median age of the patient collective) had a median QLQ-C30 of 69, whereas patients older than 71 years had a median score of 74 (p=0.784). Analysing patient's age continuously using Spearman's correlation, we found no statistically significant impact on the respective QoL (p=0.926). Regarding the preoperative performance status, we found patients with a preoperative ECOG score of 0 having a significantly higher QoL compared to patients with a preoperative ECOG score of 1 or 2 (p<0.001 respectively, Figure-1A).

#### Intraoperative characteristics

QoL for patients whose cystectomy was performed by an experienced surgeon (>100 previous radical cystectomies) had a median QLQ-C30 score of 83 whereas patients whose radical cystectomies were performed by a less experienced surgeon (<100 previous radical cystectomies) had a median QLQ-C30 score of 74 (p=0.186, Figure-1B). However, when using the predefined cut-off value, 53% (experienced) vs. 35% (less experienced) of the patients had a QLQ-C30 score of >70 (p=0.019).

A nerve-sparing radical cystectomy was performed in 77 of 152 patients (50.7%). If a nerve-sparing operation was performed, median postoperative QoL was significantly increased (QLQ-C30 82 vs. 69; p=0.001, Figure-1C). 64% of the nerve-sparing group had a QLQ-C30>70 (vs. 36%, p<0.001).

There were no statistical significances regarding intraoperative complications (44 vs. 50% QLQ-C30≥70, p=0.731), postoperative complications (49 vs. 51%, p=0.744), need for perioperative blood transfusion (36 vs. 53%, p=0.116), intraoperative blood loss (<500mL: 55% QLQ-C30≥70 vs. ≥500mL: 47%, p=0.355), and the operation duration (median operation time: 217 minutes; <217 minutes: 58% QLQ-C30≥70 vs. 43%, p=0.077).

Regarding the pathological tumour stage, patients with locally advanced disease (pT3-4) as well as patients with lymph node involvement did not have statistically significant different QLQ-C30 scores than those with limited disease as well as without lymph node involvement (p=0.760, p=0.632 respectively).

#### Postoperative time course and adjuvant therapies

Regarding the postoperative time course, we found no statistically significant differences when comparing patient's QOL after 3 months, 6 months, 9 to 12 months, 1 year, 2 to 3 years, 4 to 5 years, and more than 5 years postoperatively. In detail, patients who were analysed during their first year of follow-up had a median QLQ-C30 of 67 (39% QLQ-C30≥70); patients who were analysed after more than one year postoperatively had a median score of 75 [p=0.078 (53% QLQ-C30≥70, p=0.168)].

Adjuvant systemic chemotherapy or palliative chemotherapy due to tumour recurrence was performed in 19 of 152 patients (12.5%). Median QLQ-C30 score for those patients was 67 (vs. 82 if no chemotherapy was performed, p=0.04). Among the patients who underwent chemotherapy, 33% had a QLQ-C30 of ≥70 (vs. 52%, p=0.174). There was no significant difference if the chemotherapy was performed within the last 12 months before answering the questionnaire, or more than 12 months ago (p=0.635).

During the postoperative time course, radiotherapy was performed in 5 of 152 patients (3.9%). Median QLQ-C30 was 77 if no radiotherapy was performed, and 41 if radiotherapy was performed (p=0.006). None of the patients who underwent radiotherapy reached a QLQ-C30 score of ≥70 (p=0.02).

In total, 19 patients (12.5%) suffered from recurrence of their malignancy. There was no statistically significant difference when comparing these patients with those patients without recurring disease (41% QLQ-C30≥70 vs. 52%, p=0.462).

Patients who were symptomatic at the time of follow-up had a significantly lower QoL than those being asymptomatic (p=0.006, Figure-1D). In detail, patients suffering from weight loss had a significantly lower QoL than asymptomatic ones (p=0.003) whereas patients with night sweats as well as flank pain did not (p=0.076, p=0.214 respectively). The symptomatic as well as asymptomatic patients did not differ in terms of disease extent and lymph node involvement (p=0.800). Among the asymptomatic patients, 60% had a QLQ-C30 of ≥70 (symptomatic patients: 34%, p=0.003).

#### Functional outcome

**Figure 2:**
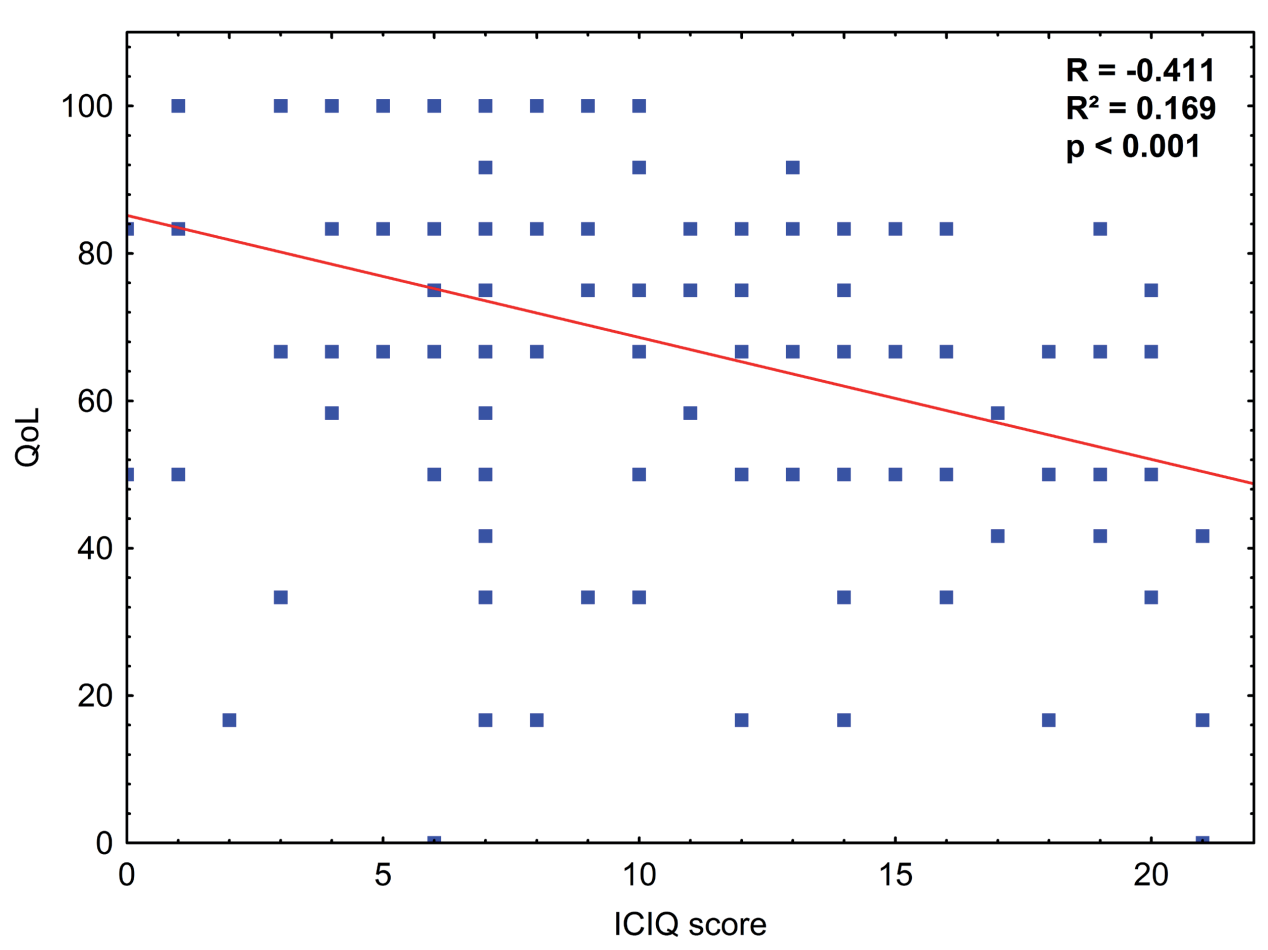
Impact of patient's ICIQ-SF score on health-related quality of life based on EORTC QLQ-C30 global health status using Spearman's rank correlation. A p value <0.05 was considered statistically significant (QOL=quality of life).

Addressing the potential impact of incontinence, we found a median QLQ-C30 of 69 for daytime incontinence and a median QLQ-C30 of 82 for daytime continence (p<0.001, Figure-1E). Impact of severity of incontinence was analysed based on daily pad usage [0-1 pad (83) vs. >2 pads (65), p<0.001, Figure-1F], and based on the ICIQ-SF [dry/mild incontinence (median QLQ-C30: 83) vs. moderate incontinence (83) vs. severe incontinence (67), p<0.001, Figure-1G]. Continuous analysis of the respective ICIQ-SF scores confirmed our primary results (p>0.001, Figure-2). Among the dry patients as well as patients with mild incontinence, 69% had a QLQ-C30 score of ≥70 (moderate incontinence: 66%, severe incontinence: 28%; p<0.001). In line, 65% of the patients in need of 0-1 pads per 24h had a QLQ-C30 score of ≥70 (2 pads: 62%, >2 pads: 30%; p<0.001). Among the patients that achieved daytime continence, 61% had a QLQ-C30 score of ≥70 (no daytime continence: 28%, p<0.001). Patients suffering from urinary urgency had a median QLQ-C30 of 70, whereas patients with no urgency symptoms had a median score of 82 (p=0.015). Regarding the predefined cut-off values, 28% of the patients suffering from urinary urgency achieved a QLQ-C30 score of ≥70 (no urgency symptoms: 55%, p=0.007).

Analysis of sexual outcome revealed a significant influence of IIEF-5 [<20 (median QLQ-C30: 75) vs. >20 (83), p=0.045, Figure-1H], but not of FSFI-6 (FSFI-6≤18 vs. FSFI>18; p=0.800). The patient subgroup that skipped the IIEF-5 questionnaire did not differ statistically significant in age (p=0.786) as well as ICIQ-SF (p=0.863) to those who answered the questionnaire. Among the patients with an IIEF-5 score of 20-25, 86% achieved a QLQ-C30 score of ≥70 (<20: 51%, p=0.078).

#### Multivariate analysis of prognostic features for health-related QoL

In a last step, a multivariate analysis was performed using a multiple logistic regression model. Hereby, all prognostic features that had statistically significant results in the univariate analysis were included. In detail, independent prognostic relevance could be confirmed for preoperative ECOG performance status of 0 (p=0.020 vs. ECOG 1, p=0.047 vs. ECOG 2), the experience of the respective surgeon (≥100 vs. <100 previous cystectomies, p=0.021), and daytime continence (p=0.032). Results of the multivariate analysis are summarized in Table-3.

**Table 3:** Multivariate analysis of prognostic features that showed significant results in univariate analysis.

Prognostic feature	P value	OR	95% CI	
Gender [male/female]	0.185	2.745	0.618	12.193
ECOG 1 vs. 0	**0.020**	0.336	0.134	0.842
ECOG 2 vs. 0	**0.047**	0.121	0.015	0.974
ICIQ group mild vs. moderate	0.394	1.76	0.48	6.449
ICIQ group mild vs. severe	0.382	0.504	0.109	2.342
Surgeon experience <100 vs. >100 prev. rCx [y/n]	**0.021**	3.442	1.203	9.847
Nerve-sparing [y/n]	0.165	2.029	0.747	5.511
Radiation therapy [y/n]	0.999	0	0	.
Symptoms [y/n]	0.109	0.458	0.176	1.192
Urge [y/n]	0.259	0.486	0.139	1.702
Daytime continence [y/n]	**0.032**	3.131	1.101	8.908
Daily pad usage 2 vs. 0-1	0.817	0.859	0.238	3.105
Daily pad usage >2 vs. 0-1	0.995	0.996	0.274	3.616

**CI** = confidence interval; **ECOG** = Eastern Cooperative Oncology Group performance status score; **ICIQ** = International Consultation on Incontinence Questionnaire; **OR** = odds ratio; **RCX** = radical cystectomy.

A p value >0.05 was considered statistically signifcant.

## DISCUSSION

Orthotopic ileal neobladder has become a widespread treatment of muscle-invasive bladder cancer. Certainly, complication rates are still high and functional outcomes may be unfavourable even in high-volume centres (15–18). As a consequence, recently more attention was drawn towards health-related QoL after radical cystectomy (6). However, measurement of QoL remains difficult and requires specific tools to address health-related QoL aspects adequately. A various number of different existing instruments-generic, cancer specific and bladder cancer specific-complicate the comparison and integration of the pre-existing literature (19). We used the validated EORTC QLQ-C30 that has been frequently used in multiple studies investigating QoL after radical cystectomy (20–27). In recent years, many efforts have been made to define certain cut-off values in order to select those patients with unmet needs for further support. Snyder et al. evaluated more than 500 oncologic patients and found a QLQ-C30 global health status cut-off value of 70 to have a sensitivity of up to 86% and a negative predictive value of up to 94% (14, 28). The aim of the current study was to evaluate various potential prognostic features on patients QoL after orthotopic urinary diversion, focusing on preoperative, perioperative as well as postoperative features. To allow a better comparison with existing literature, we analysed the postoperative QLQ-C30 global health status as a continuous parameter as well as following Snyder et al. (14).

Addressing patient-derived features, we found no statistically significant impact of preoperative patient's age. This finding is in line with recent publications indicating that radical cystectomy with orthotopic urinary diversion can be safely performed even in elderly patient cohorts (29). Imbimbo et al. even found significant increased QLQ-C30 scores in patients older than 65 years (30). However, there is also evidence that elderly patients are still less likely to receive orthotopic urinary diversion (31). Going further, we found a significant impact of the preoperative performance status on postoperative QoL in univariate as well as in multivariate analysis. Given the fact that health-related QoL measurements take into consideration multiple aspects including physical status, general health and social interaction, it seems intuitive that patients with a better performance status profit more from a procedure that aims to allow an unaltered, active lifestyle. In line, it has been shown that patients undergoing orthotopic urinary diversion are more active and have a better physical function compared to patients undergoing incontinent urinary diversion (32). Our observations indicate that age per se is not associated with impaired QoL after orthotopic urinary diversion and discussion of QoL aspects prior to radical cystectomy should be based on the patient's performance status rather than on the respective patient's age. Regarding preoperative patient selection, our results indicate, that elderly patients who have an adequate performance status, can be treated with an orthotopic urinary diversion and still have a good postoperative QoL. Since radical cystectomy for octogenarians becomes more accepted, this finding has important clinical implications (33).

In the current study, various intraoperative characteristics on the patient's postoperative QoL were analysed, indicating a significant impact of the surgeon's experience both in univariate as well as in multivariate analysis. To our knowledge, evidence regarding the surgeon's learning curve impact on patients postoperative QoL after radical cystectomy and orthotopic urinary diversion is still very limited. However, there is evidence that excellent functional outcomes can be achieved in high-volume centers (18). In line, it has been shown that the surgeon's individual learning curve has a significant impact on functional outcome after radical prostatectomy and is therefore directly affecting the patient's postoperative QoL (34). The impact of surgeon's experience on postoperative QoL after orthotopic urinary diversion might highlight the importance of the referral to high-volume centers and therefore has clinical implications.

Addressing a potential time course of patient's QoL after orthotopic urinary diversion, we found no statistically significant differences in QoL after the respective moments of follow-up. However, there was a statistical trend favouring those patients who were evaluated more than 1 year postoperatively. This might indicate that QoL increases during the postoperative course of time as described by Kulaksizoglu and colleagues (27). We found that, if patients underwent adjuvant or palliative chemotherapy or radiotherapy, QoL was significantly lower, no matter if the respective therapy was performed within 1 year before answering the questionnaires or not. It has to be stated, however, that there might be a relevant non-responder bias in our patient cohort. Thus, these findings have to be interpreted with caution. Our results are partly in line with the findings of Cognetti et al. who also found a decline of QoL after initiation of adjuvant Cisplatin-based chemotherapy. However, the authors stated that there was a subsequent improvement after the initial decline of QoL, which is not reflected by our data (35).

To assess the impact of urinary incontinence on health-related QoL, we used the validated ICIQ-SF questionnaire and pad usage and found a significant impact of urinary leakage. These findings are in line with the results of Takenaka et al. who evaluated long-term outcome in QoL after orthotopic neobladder in 86 patients and found worse QoL scores in patients with daytime incontinence (36). Zahran et al. analyzed QoL of 74 women after radical cystectomy and orthotopic neobladder using QLQ-C30 and observed a significant negative impact of incontinence (7). In line with Imbimbo et al., we found urinary incontinence being an independent prognostic feature of an impaired QoL (30). However, the findings of the current study addressing the great impact of continence are based on multiple consistent parameters in a contemporary patient collective and therefore add important information to the existing literature.

It is commonly accepted that a significant proportion of patients after orthotopic bladder replacement suffer from sexual dysfunction (37). In our patient collective, for instance, only 5.6% of the men could be classified as “no sexual dysfunction” based on the respective IIEF-5 score (>20). Since no preoperative data was available, these results have to be interpreted with caution. However, we were able to show that health-related QoL was significantly increased in those patients who had a postoperative IIEF-5 score of more than 20. To date, there is only limited data concerning the impact of sexual function on QoL after radical cystectomy. Studies from large prostate cancer series, on the other hand, consistently report better QoL outcomes for nerve-sparing radical prostatectomies indicating a major impact of sexual preservation on QoL aspects after major pelvic surgery (38). Summarizing, our findings highlight the great impact of functional aspects on health-related QoL. However, it is somewhat difficult to imply the important role that postoperative continence plays for health-related QoL into daily clinical practice. Naturally, every surgeon tries to achieve the best possible functional result. However, one might focus on the development of continence-preserving technical modifications. Regarding radical prostatectomy for example, the posterior rhabdomyosphincter reconstruction, also known as Rocco stitch, has been proposed to improve postoperative continence (39, 40). Additionally, we still lack knowledge how the shape of the neobladder (e.g. U- vs. W-shape) affects postoperative continence recovery.

Certainly, our study is not devoid of limitations. Major drawback is its retrospective nature with all the known limitations that are inherent. The current study is a cross-sectional study and QoL has not been measured at specific time-points. Nevertheless, the current study provides QoL analyses from various different follow-up periods, providing a possible idea of the natural process of QoL after radical cystectomy. In the current study, the EORTC QLQ-C30 global health status was used. It has to be stated, that, unlike the EORTC QLQ-BLM30, this questionnaire is not bladder cancer specific and lacks additional information regarding body image, urostomy problems, and use of catheters. The QLQ-BLM30 has been used in several publications before, but is still not validated (23, 26, 41). Additionally, the Functional Assessment of Cancer Therapy-General, Bladder (FACT-BL) and Vanderbilt Cystectomy Index (FACT-VCI) are validated bladder cancer specific questionnaires that have been used in health-related QoL studies before (42). However, in a recent meta-analysis including data from 18 studies that investigated health-related QoL after radical cystectomy, 10 out of 18 used the EORTC QLQ-C30 questionnaire (43). This indicates, that, despite being not cancer specific, the QLQ-C30 questionnaire is well established in QoL analyses of patient cohorts that underwent radical cystectomy and orthotopic urinary diversion.

## CONCLUSIONS

The current study analyses the impact of multiple potentially prognostic features on QoL after orthotopic urinary diversion. We report health-related QoL outcomes in a contemporary patient cohort. Hereby, we were able to show that preoperative ECOG status, surgeon experience and daytime incontinence are independent prognostic features for a good postoperative QoL.

## ABBREVIATIONS

QoL: = Quality of life
QLQ-C30: = Quality of life questionnaire C30
EORTC: = European Organization for Research on Treatment of Cancer
ICIQ-SF: = International Consultation on Incontinence Questionnaire short-form
IIEF-5: = International Index of Erectile Function
FSFI-6: = Female Sexual Function Index (abridged)
FACT-BL: = Functional Assessment of Cancer Therapy-General, Bladder
FACT-VCI: = Vanderbilt Cystectomy Index
